# Analysis of the Clinicopathological Characteristics, Genetic Phenotypes, and Prognostics of Primary Pulmonary and Bronchial Adenoid Cystic Carcinoma

**DOI:** 10.1111/1759-7714.15526

**Published:** 2025-01-19

**Authors:** Zhengyang Hu, Xing Jin, Jian Wang, Qihai Sui, Yanjun Yi, Dejun Zeng, Zhencong Chen, Qun Wang, Jiacheng Yin, Lin Wang, Zongwu Lin

**Affiliations:** ^1^ Department of Thoracic Surgery, Zhongshan Hospital Fudan University Shanghai China; ^2^ Department of Thoracic Surgery Shanghai Xuhui Central Hospital Shanghai China

**Keywords:** clinicopathological characteristics, genetic phenotypes, primary pulmonary and bronchial adenoid cystic carcinoma, prognosis

## Abstract

**Background:**

Primary pulmonary and bronchial adenoid cystic carcinoma (PACC) is a rare, low‐grade malignant tumor of the lung. However, the relationship between its clinical features, surgical prognosis, and genetic phenotype has not been fully described.

**Methods:**

PACC patient information was collected from the SEER, TCGA, and Zhongshan Hospital, Fudan University (FDZSH) databases. Overall survival (OS) was evaluated using the Kaplan–Meier method. Univariate and multivariate analyses through Cox proportional hazards regression identified risk factors that predicted OS. The limma and matfools packages from R were used to compare the differential genes and mutations between PACC and LUAD, respectively.

**Results:**

Two hundred and ninety‐two patients, 14 patients, and 12 patients with PACC were identified from the SEER, TCGA, and FDZSH databases, respectively. The 3‐, 5‐, and 10‐year OS of the PACC patients were 91.7%, 88.6%, and 85.0%, respectively, compared to 95.8%, 93.9%, and 93.3% for patients who underwent surgery. Race, pathological grade, M stage, regional node examination, and regional node positive were independent prognostic factors for the OS of patients who underwent surgery. The gene map of PACC and lung adenocarcinoma (LUAD) shows significant differences. Common mutations found in lung cancer were almost undetectable in PACC patients, whereas mutations in the NOTCH pathway were more common. TMB levels and PD‐1/PD‐L1 expressions were also lower in PACC patients.

**Conclusion:**

Our study analyzed the main factors that influence the prognosis of PACC patients undergoing surgery and discovered the unique genetic phenotype of PACC.

## Graphical abstract

PACC patient information was collected from the SEER, TCGA, and Zhongshan Hospital, Fudan University (FDZSH) databases. We focused on the factors affecting the prognosis of PACC patients who underwent surgery and constructed a nomogram. In addition, we revealed the molecular signature of PACC.

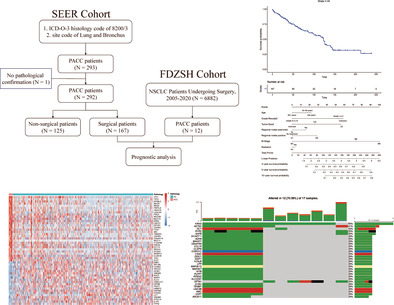

## 1 Background

Adenoid cystic carcinoma (ACC) is a rare secretory gland malignancy, usually occurring in the salivary glands of the head and neck, and accounting for 1% of all head and neck cancers [1]. It can also occur in the lacrimal and ceruminous glands, as well as other sites, including the nasal and paranasal sinuses, trachea, lung, breast, and larynx [2, 3, 4].

Primary pulmonary and bronchial adenoid cystic carcinoma (PACC) is a rare, low‐grade malignant tumor of the lung, classified as a salivary gland‐type tumor, together with mucoepidermoid carcinoma (MEC) and epithelial myoepithelial carcinoma (EMC). It accounts for about 0.09%–0.2% of all primary lung cancers [5]. Since PACC originates from the submucosal glands in the tracheobronchial tree, which are more densely distributed in the large airways than in the small airways, the tumor often occurs in the trachea and hilus of the lung. Previous studies have shown that about 90% of PACCs occur in the trachea and hilus [6] and account for 10% of bronchial tumors [7]. As a result, patients usually present with upper respiratory symptoms, such as wheezing, dyspnea, or hemoptysis [8].

Surgery is the preferred treatment for PACC, and early‐stage patients can significantly benefit from surgery. It is also a radiotherapy‐sensitive tumor. Radiotherapy is an option for patients with postoperative pathology suggestive of positive margins or recurrence who are unable to undergo a second surgery [9].

To date, research on PACC has been limited to case reports or small case series, and its clinical features, molecular characteristics, treatment, and outcomes are not well understood. Large‐scale case summaries will contribute to a better understanding of the disease and improve diagnosis and treatment. In our study, we retrospectively analyzed the clinical features, prognosis, and possible prognostic factors of 292 PACC patients in the SEER database, specifically 167 patients who underwent surgery, and 12 patients who underwent surgery at Zhongshan Hospital, Fudan University (FDZSH). The TCGA database was also used to explore the molecular characteristics of PACC.

## 2 Patients and Methods

### 2.1 Patient Selection

In our current study, a total of 292 patients with PACC from the SEER database were included, among them 167 patients underwent surgery. Pulmonary and bronchial ACC patients were classified according to the International Classification of Diseases for Oncology, third edition (ICD‐O‐3) histology code 8200/3 and site code for Lung and Bronchus.

Twelve patients who underwent surgery at FDZSH, and had postoperative pathologically confirmed PACC between 2005 and 2020 were also included in the study.

We collected patient characteristics (age at diagnosis, sex, and race); cancer characteristics (primary site, tumor size, pathological grade, and TNM stage); treatment details (records of surgery, radiation therapy, and chemotherapy); and follow‐up records (cause of death, cancer‐specific deaths, and overall survival) from the SEER and FDZSH databases. The TNM stages were classified according to the eighth edition criteria of the American Joint Committee on Cancer (AJCC).

**FIGURE 1:**
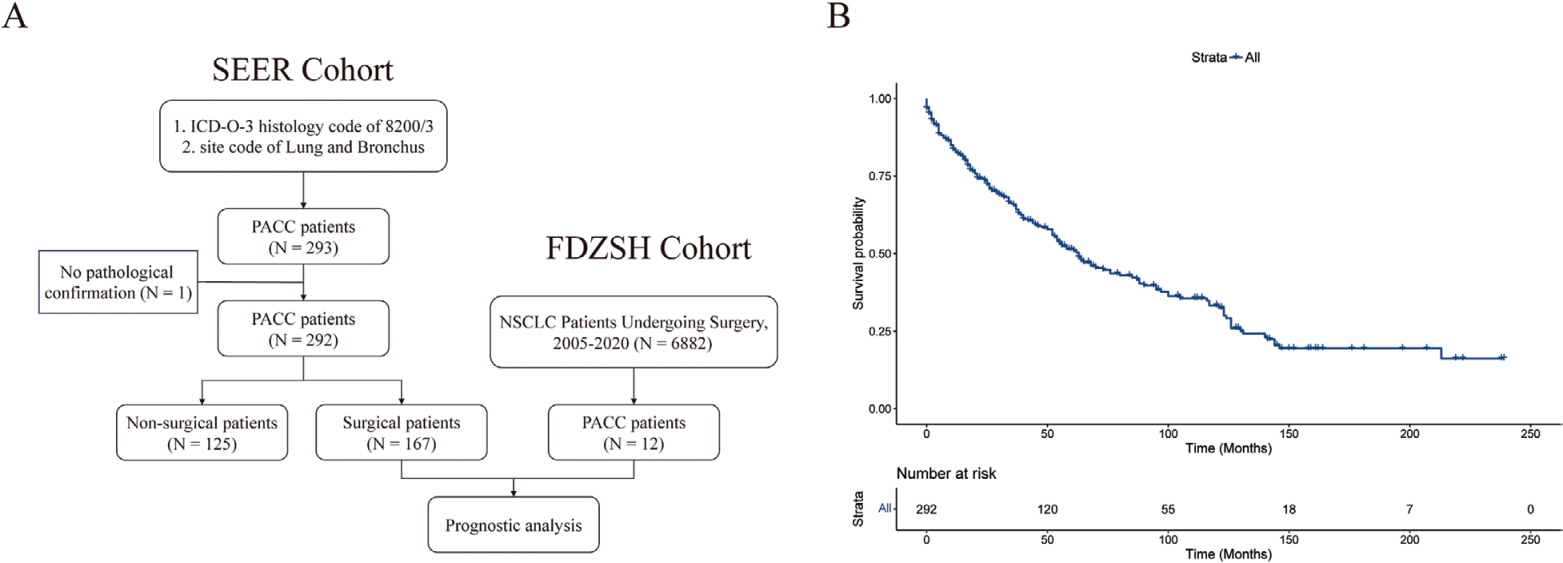
Flowchart of different database screening methods (A) and overall survival of 292 primary pulmonary and bronchial adenoid cystic carcinoma patients from the SEER database (B).

All 292 patients from the SEER database were analyzed in the overall analysis. In addition, 167 of these patients who underwent surgery from the SEER database and 12 from FDZSH underwent postoperative patient survival analysis to understand the factors that influence the prognosis of surgical patients. The specific screening method is shown in the flowchart in Figure 1A.

Fourteen PACC patients and 488 lung adenocarcinoma patients from the TCGA database were included for genetic analysis. We downloaded their gene expression and mutation data from the TCGA database for differential gene and mutation analysis.

### 2.2 Statistical Analysis

Continuous variables are presented as the mean with SD, and categorical variables are presented as numbers and percentages. Survival analyses were performed using R (Version 4.2.0) and the survival package. The Kaplan–Meier method and the log‐rank test were used to construct and compare survival curves. Potential factors affecting survival were explored using Cox regression model analysis. Factors with a *p* < 0.1 in univariate analysis were included in multivariate analysis. To exclude the effect of highly correlated variables, such as T stage and tumor size, on the accuracy of survival analysis, we assessed the accuracy of the regression model using the mean‐squared error and coefficient of determination. After combining the accuracy from the multivariate analysis with the clinical relevance of the variables, we selected candidate variables for inclusion in the multivariate regression analysis to analyze their impact on prognosis. The key and fundamental principle for screening candidate variables for multivariate regression analysis was to include baseline variables that were considered clinically relevant, or those that showed a univariate relationship with the outcome in the multivariate regression model. The construction of the nomogram was based on the results of the multivariate analysis, using the rms package. The lms function was utilized to construct the fitted predictive model, and subsequently, the nomogram function was used to construct the nomogram. At the same time, the stability and accuracy of the model need to be considered in the construction process. Subsequently, stepwise regression was applied to determine the appropriate scale score for each variable. The above analysis process was implemented using the R language rms package. *p* values less than 0.05 were considered statistically significant.

### 2.3 Genomic Analysis

Differential gene analysis was performed using the R package limma. First, we constructed a design matrix based on the sample classification information, which was used to fit the linear model. Subsequently, genetic differences between PACC and lung adenocarcinoma were designated for comparison. The linear model was then fitted to the data using the design matrix, and the matrix was applied to the fitted model to obtain the contrast coefficients. Finally, after converting the fitted model into an empirical Bayes model, we obtained a more reliable estimate of differential expression. The thresholds for significant genes were adjusted *p* < 0.05 and |fold change| > 1. Based on the differential expression results, we visualized them using volcano plots or heatmaps to explore the overall structure and grouping of samples. After obtaining the differential genes, we performed enrichment analysis using Metascape (http://metascape.org/gp/index.html#/main/step1) to gain insights into the biological processes and pathways. The maftools package was used to analyze differentially mutated genes in PACC and other LUAD, as well as the frequency of mutations. After loading the MAF file containing the mutation information and classification data for the samples, we first explored the data by examining the summary statistics. We then used the oncoplot function to generate an oncoplot, which is a visual representation of mutation data across samples and genes. The oncoplot displays the tumor mutation burden (TMB) at the top, indicating the number of somatic mutations per megabase of the genome. The middle section shows the mutation status of each gene across samples, with different colors representing different mutation types. The right bar plot displays the proportion of samples containing mutations in each gene, as well as the composition of mutation types. Finally, CIBERSORTx (https://cibersortx.stanford.edu/) was used to reverse‐convolutionally calculate the proportion of immune cells in the tumor microenvironment. The code used for the analysis is shown in Supplementary File 2.

## 3 Results

### 3.1 Clinical Features

We screened a total of 292 patients with PACC from the SEER database, and after excluding one patient with no clear pathology, a total of 292 patients were included in the study. The demographic and clinical data of the overall population are summarized in Table 1. Males represented 47.3% of the cohort, and the average age was 61.68 ± 14.90 years. Whites accounted for 81.8%. In 27.1% of patients, the tumor was located in the main bronchus. Another 28.8%, 6.16%, and 21.7% of tumors were located in the upper, middle, and lower lobes, respectively. According to the eighth edition of AJCC lung cancer staging, there were 82, 40, 58, and 54 patients with Stages I, II, III, and IV, respectively. In terms of treatment received, more than half of the patients received surgery or a surgical‐based multimodality therapy (167, 57.2%), whereas another 8, 38, and 18 patients received chemotherapy, radiotherapy, or chemoradiotherapy. The 3‐, 5‐, and 10‐year OS of the overall population were 91.7%, 88.6%, and 85.0%, respectively, with a median OS (mOS) of 63 months (Figure 1B).

**TABLE 1:** Baseline characteristics of primary pulmonary and bronchial adenoid cystic carcinoma patients in the SEER database.

	SEER cohort
Characteristics	***N =*** 292
Age (years), median (IQR)	61.68 (46.78–76.58)
Sex	
Female	154 (52.7%)
Male	138 (47.3%)
Race	
Asian or Pacific Islander	22 (7.53%)
Black	31 (10.6%)
White	239 (81.8%)
Primary site	
Main bronchus	79 (27.1%)
Upper lobe, lung	84 (28.8%)
Middle lobe, lung	18 (6.16%)
Lower lobe, lung	81 (27.7%)
Lung, NOS	30 (10.3%)
Laterality	
Left	143 (49.0%)
Right	137 (46.9%)
Unknown	8 (2.74%)
Grade	
Grade I or II	66 (22.6%)
Grade III or IV	21 (7.19%)
Unknown	205 (70.2%)
Tumor size	36.42 (11.89–60.95)
T stage	
T1	70 (24.0%)
T2	62 (21.2%)
T3	26 (8.90%)
T4	63 (21.6%)
Tx	71 (24.3%)
N stage	
N0	167 (57.2%)
N1	36 (12.3%)
N2	27 (9.25%)
N3	10 (3.42%)
Nx	52 (17.8%)
M stage	
M0	193 (66.1%)
M1	54 (18.5%)
Mx	45 (15.4%)
Stage	
I	82 (28.1%)
II	40 (13.7%)
III	58 (19.9%)
IV	54 (18.5%)
Unknown	58 (19.9%)
Surgery	
Yes	167 (57.2%)
None/unknown	125 (42.8%)
Radiation	
None/unknown	183 (62.7%)
Yes	109 (37.3%)
Chemotherapy	
No/unknown	241 (82.5%)
Yes	51 (17.5%)

### 3.2 Clinical Features of Patients Undergoing Surgery

A total of 167 and 12 patients underwent surgery in the SEER and FDZSH databases. The demographic and clinical data are summarized in Table 2. For the SEER database, the average age was 58.05 ± 14.27 years, and females accounted for 50.3% of the total. In 25.1% of patients, the tumor was located in the main bronchus, and the rest were located in the lungs. In addition, there were 57, 35, 37, and 9 patients with postoperative Stages I, II, III, and IV, respectively. Besides surgery, 66 patients (39.5%) were treated with radiotherapy or chemotherapy. The mean tumor size was 36.65 ± 21.54 mm. One hundred and forty patients were examined for regional nodes, with a mean number of 10.40 ± 8.97, and 53 of these patients had positive nodes.

**TABLE 2:** Baseline characteristics of PACC patients who underwent surgery in the SEER and FDZSH databases.

	SEER cohort	FDZSH cohort
	*N* = 167	*N* = 12
Age	58.05 (43.78–72.32)	57.17 (48.38—65.96)
Sex		
Female	84 (50.3%)	6 (50.0%)
Male	83 (49.7%)	6 (50.0%)
Race		
Asian or Pacific Islander	12 (7.19%)	12 (100.0%)
Black	15 (8.98%)	
White	140 (83.8%)	
Smoking history	—	
Yes		4 (33.3%)
No		8 (66.7%)
Primary site		
Main bronchus	42 (25.1%)	6 (50.0%)
Upper lobe, lung	50 (29.9%)	2 (16.7%)
Middle lobe, lung	9 (5.39%)	3 (25.0%)
Lower lobe, lung	53 (31.7%)	1 (8.33%)
Lung, NOS	13 (7.78%)	
Grade ecode		—
Grade I or II	54 (32.3%)	
Grade III or IV	14 (8.38%)	
Unknown	99 (59.3%)	
Laterality		
Left	95 (56.9%)	2 (16.7%)
Right	70 (41.9%)	6 (50.0%)
Unknown	2 (1.20%)	4 (33.3%)
T stage		
T1	50 (29.9%)	3 (25.0%)
T2	43 (25.7%)	7 (58.3%)
T3	17 (10.2%)	1 (8.33%)
T4	27 (16.2%)	1 (8.33%)
TX	30 (18.0%)	
N stage		
N0	101 (60.5%)	8 (66.7%)
N1	31 (18.6%)	3 (25.0%)
N2	15 (8.98%)	1 (8.33%)
N3	1 (0.60%)	
NX	19 (11.4%)	
M stage		
M0	135 (80.8%)	12 (100%)
M1	9 (5.39%)	
MX	23 (13.8%)	
Stage		
I	57 (34.1%)	4 (33.3%)
II	35 (21.0%)	7 (58.3%)
III	37 (22.2%)	1 (8.33%)
IV	9 (5.39%)	
Unknown	29 (17.4%)	
Residual tumor	—	
R0		11 (91.7%)
R1		1 (8.33%)
Radiation		
No	114 (68.3%)	10 (83.3%e)
Yes	53 (31.7%)	2 (16.7%)
Chemotherapy		
No	142 (85.0%)	8 (66.7%)
Yes	25 (15.0%)	4 (33.3%)
Tumor size	36.65 (15.11–58.19)	3.35 (1.85–4.85)
Regional nodes examined	10.40 (1.43–19.37)	14.7 (4.3–25.1)
No	27 (16.2%)	2 (16.7%)
Yes	140 (83.8%)	10 (83.3%)
Regional nodes positive		
N+	53 (31.7%)	4 (33.3%)
N0	87 (52.1%)	6 (50.0%)
No examined	27 (16.2%)	2 (16.7%)

The mean age of the 12 patients in the FDZSH database was 57.17 years, with a 50/50 split between males and females. Four of the patients had a history of smoking. In half of these patients, the tumor was located in the main bronchus, a higher percentage than in the SEER database. Cancer involvement is still visible at the tracheal margin in one patient (R1 resection). Four patients had recurrence or metastasis, and three patients died from their tumors. Two of the four patients received radiochemotherapy after experiencing recurrence or metastasis, and one patient with localized anastomotic recurrence underwent fiberoptic bronchoscopy tumor resection.

### 3.3 Survival of Patients Undergoing Surgery

**FIGURE 2:**
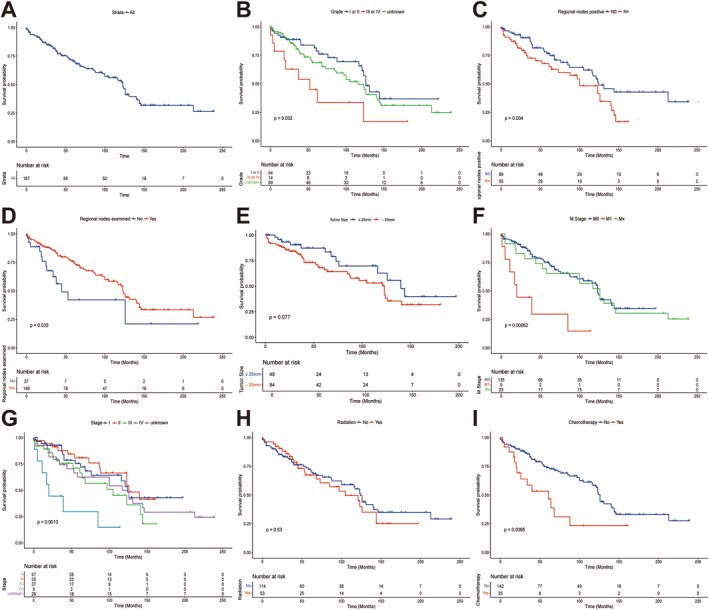
Comparison of overall survival between different groups in 167 primary pulmonary and bronchial adenoid cystic carcinoma patients who underwent surgery from the SEER database. We selected variables for presentation based on the magnitude of their impact on survival combined with clinical significance: (A) all patients, (B) grade, (C) regional nodes examined, (D) regional nodes positive, (E) tumor size, (F) M stage, (G) AJCC 8th Stage, (H) radiation, and (I) chemotherapy.

The 3‐, 5‐, and 10‐ OS of the surgery population were 95.8%, 93.9%, and 93.3%, respectively, with a mOS of 123 months. The univariate Cox regression analysis showed that age, sex, primary site, laterality, and tumor size had no significant effects on the OS of surgery patients (*p* > 0.050), whereas race, pathological grade, tumor stage, especially the M stage, and treatment methods had significant effects on OS (*p* < 0.050) (Figures 2 and S1). Interestingly, we found that whether lymph nodes were examined or not had an impact on survival (*p* = 0.036), whereas the specific number of nodes had no significant effect. As for lymph node metastases, it was clear that patients with metastases had a worse prognosis (*p* = 0.038), but it was not related to the number of metastatic nodes (*p* = 0.128). Multivariate Cox regression analysis showed that race, pathological grade, M stage, regional node exam, and regional node positive were independent prognostic factors for the OS of patients who underwent surgery (*p* < 0.050). In contrast, tumor size and whether patients received radiotherapy or chemotherapy did not affect the prognosis (Table 3).

**TABLE 3:** Univariate and multivariate Cox proportional hazards analysis of PACC patients who underwent surgery.

	Univariate analysis		Multivariate analysis	
Independent variables	HR (95% CI)	*p*	HR (95% CI)	*p*
Age (years)				
≤ 44		Reference		
45–64	0.95 (0.48–1.89)	0.884		
65+	1.72 (0.86–3.44)	0.125		
Sex				
Female		Reference		
Male	1.06 (0.67–1.68)	0.806		
Race				
Asian or Pacific Islander		Reference		Reference
Black	0.54 (0.2–1.46)	0.226	0.95 (0.33–2.74)	0.921
White	0.33 (0.16–0.68)	0.003	0.43 (0.19–0.95)	0.037
Primary site				
Main bronchus		Reference		
Upper lobe, lung	2.02 (0.81–5.01)	0.131		
Middle lobe, lung	1.16 (0.65–2.06)	0.616		
Lower lobe, lung	0.52 (0.12–2.20)	0.374		
Lung, NOS	0.72 (0.39–1.34)	0.303		
Laterality				
Right		Reference		Reference
Left	0.63 (0.40–1.01)	0.054	0.65 (0.4–1.07)	0.090
Unknown	1.00 (0.14–7.35)	0.999	1.1 (0.14–8.99)	0.926
Grade				
Grade I or II		Reference		Reference
Grade III or IV	2.79 (1.27–6.16)	0.011	3.75 (1.59–8.84)	0.003
Unknown	1.33 (0.78–2.26)	0.294	1.19 (0.65–2.15)	0.574
Tumor size (mm)				
≤ 25		Reference		Reference
> 25	1.85 (0.93–3.69)	0.080	1.16 (0.58–2.33)	0.672
T stage				
T1		Reference		
T2	1.68 (0.82–3.43)	0.158		
T3	2.08 (0.82–5.33)	0.125		
T4	3.13 (1.44–6.83)	0.004		
Tx	1.90 (0.92–3.92)	0.084		
N stage				
N0		Reference		
N1	1.23 (0.66–2.30)	0.506		
N2	1.91 (0.92–3.99)	0.084		
N3	7.57 (1.01–56.8)	0.049		
Nx	1.83 (0.98–3.42)	0.059		
M stage				
M0		Reference		Reference
M1	4.28 (1.91–9.57)	< 0.001	4.05 (1.68–9.78)	0.002
Mx	1.09 (0.62–1.94)	0.759	0.52 (0.17–1.56)	0.240
Stage				
I		Reference		
II	0.91 (0.44–1.88)	0.808		
III	1.66 (0.84–3.25)	0.142		
IV	4.98 (2.04–12.15)	< 0.001		
Radiation				
None/unknown		Reference		
Yes	1.17 (0.72–1.92)	0.526		
Chemotherapy				
No/unknown		Reference		
Yes	2.22 (1.23–4.00)	0.008		
Therapy				
Surgery		Reference		Reference
Surgery combined Radiation or chemotherapy	1.68 (1.05–2.69)	0.030	1.51 (0.90–2.54)	0.119
Regional nodes examined				
No		Reference		Reference
Yes	0.53 (0.3–0.96)	0.036	0.1 (0.03–0.33)	< 0.001
Regional nodes positive				
N0		Reference		Reference
N+	1.69 (1.03–2.78)	0.038	2.17 (1.26–3.73)	0.005
No examined	1.46 (0.67–3.18)	0.338	0.16 (0.04–0.64)	0.009

Furthermore, we have constructed a nomogram based on the SEER data to predict the 3‐, 5‐, and 10‐year survival rates. The nomogram can provide patients with personalized predictions based on the patient's specific situation, help doctors understand the patient's condition and prognosis, and develop a more appropriate treatment plan. The *c*‐index of this nomogram was 0.681 (95% CI: 0.610–0.725) (Figure S1).

### 3.4 Genetic Analysis

**FIGURE 3:**
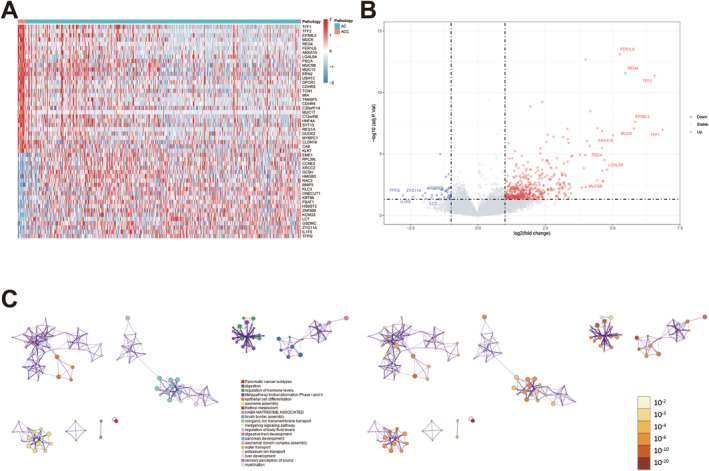
Differences in gene expression between PACC and LUAD. (A, B) Heatmap and volcano map of differentially expressed genes between PACC and LUAD. (C) Enrichment analyses of differentially expressed genes between PACC and LUAD.

In the DEG analysis, we selected 14 PACC patients to compare with 488 LUAD patients from the TCGA database. We obtained a total of 504 differential genes, of which 463 were upregulated and 41 downregulated in PACC patients. Heatmaps and volcano plots demonstrated the differential genes between PACC and LUAD (Figure 3A,B) Subsequently, we performed enrichment analyses using the above differential genes. The results of the enrichment analysis are presented in Figure 3C.

**FIGURE 4:**
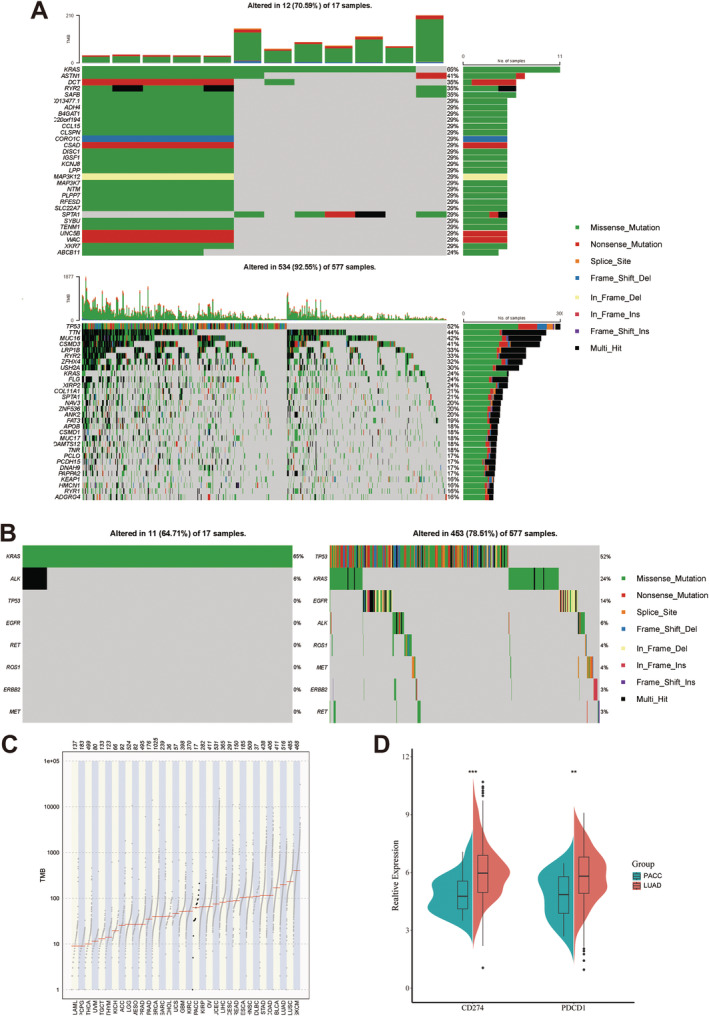
Differences in gene mutations between PACC and LUAD. (A) Gene mutations in PACC (up) and LUAD (down) patients. (B) Common gene mutations in lung cancer in PACC (left) and LUAD (right) patients. (C) Tumor mutation burden in PACC patients. (D) PD‐1/PD‐L1 expression in PACC and LUAD patients.

We then analyzed the PACC and LUAD gene mutation data. Mutations in PACC are predominantly KRAS, whereas TP53, TTN, and MUC16 mutations are less common. In addition, apart from KRAS, only one patient was detected with an ALK fusion, and no mutations were detected in the rest of the common driver genes of LUAD, such as EGFR, MET, and ROS1 (Figure 4A,B) Similarly, these eight common mutations were also not detected in the 12 FDZSH patients. TMB levels and PD‐1/PD‐L1 expressions were also lower in PACC patients (Figure 4C,D).

**FIGURE 5:**
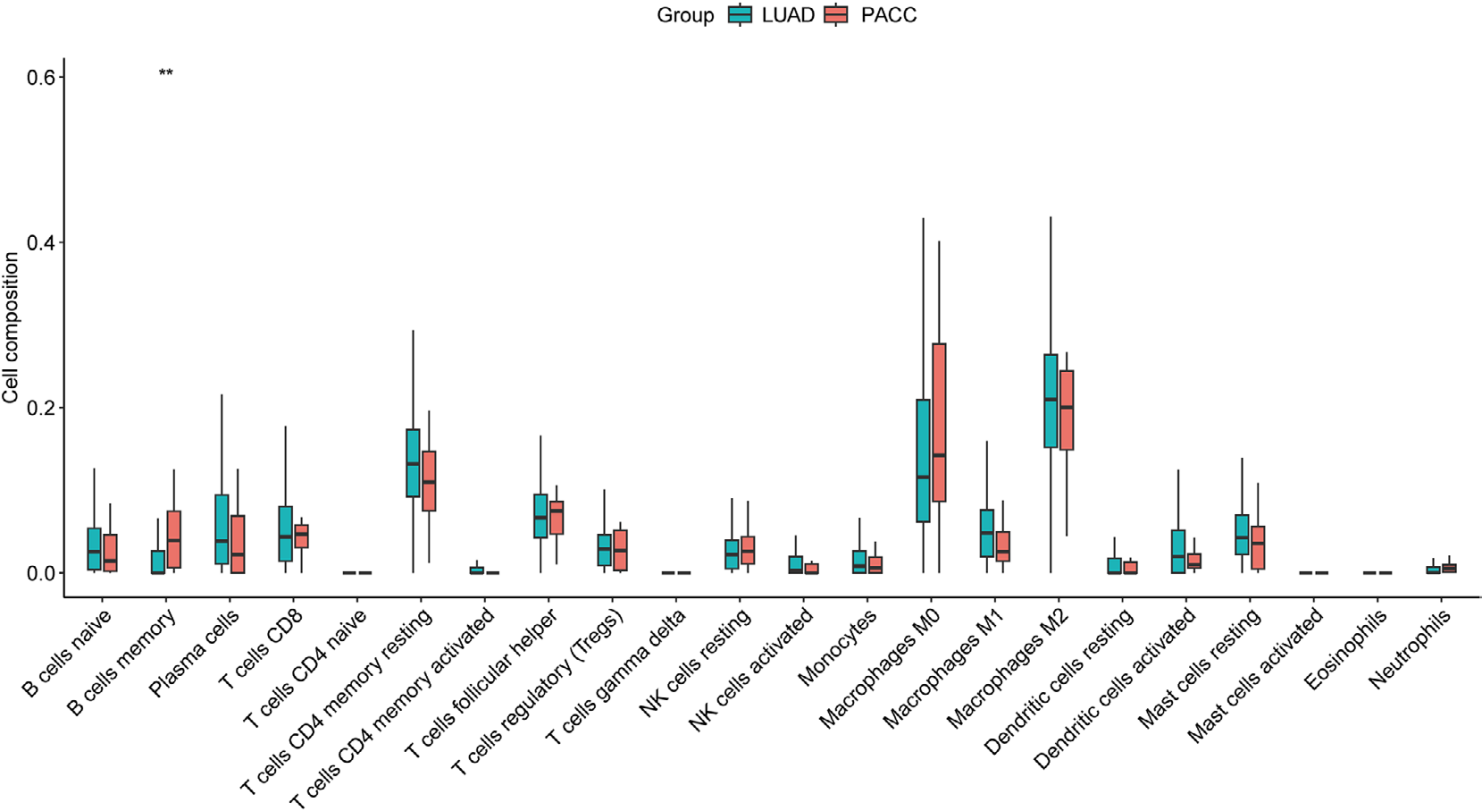
Composition of different immune cells in the PACC and LUAD tumor microenvironment.

We subsequently deconvoluted the TCGA data using CibersortX and found that the composition of the immune microenvironment was similar in PACC and LUAD, with only memory B‐cell infiltration elevated in PACC (Figure 5).

## 4 Discussion

In the current study, we have summarized the clinical and molecular features of PACC. PACC is a rare primary malignant tumor of the lungs. We used the SEER database to study the clinical characteristics and prognostic indicators of PACC. Gender was not associated with its morbidity, and it is more densely distributed in the bronchial region. Patient survival was relatively good for PACC, with the 10‐year survival reaching 85.0% for all patients, and 93.3% for surgical patients. This is similar to previous reports and better than other types of lung cancer [10]. Race, pathological grade, M stage, regional node examination, and regional node positive were independent prognostic factors for survival. In contrast, the tumor size, the number of lymph nodes examined, and the number of positive lymph nodes were not associated with prognosis. This may be because PACC is less malignant and less likely to spread to the lymph nodes than other kinds of lung cancer [7]. Interestingly, we found that the prognosis of patients who received chemotherapy was rather worse, which may be related to the late stage of these patients and the lost opportunity for surgery. Additionally, previous studies also indicate that PACC may not be sensitive to chemotherapy. Chemotherapy is also not its first line of treatment and is only recommended as palliative chemotherapy for patients with symptoms and/or a high tumor burden [11]. To date, there is no widely accepted staging system for PACC. Most of the studies, including our current study, have applied lung cancer staging methods to PACC. He et al. performed a prognostic analysis of bronchial tumors, including PACC, using the SEER database [12]. Wang et al. performed a prognostic analysis of 191 patients with PACC [13]. Combining their study with our results, we propose that tumor size, pathological grade, lymph node metastasis, and distant metastasis can be used as criteria for staging PACC.

PACC is a low‐grade malignant tumor, and early diagnosis of PACC is a challenge due to its atypical symptoms. Wang et al. summarized the early clinical symptoms of PACC, with cough and dyspnea being the most common presentations [13]. This may be related to its original site. Therefore, in patients with chronic irritant cough with paroxysmal exacerbation, a chest CT is necessary.

Surgery is the preferred treatment for PACC, and early‐stage patients can benefit significantly after undergoing surgery. Our analysis of the SEER database showed that the 10‐year survival rate for PACC patients who underwent surgery was up to 93.3% after surgery, with a median survival time of 123 months. PACC is a radiotherapy‐sensitive tumor. Due to its prevalence in the trachea and main bronchus, complete surgical resection is difficult. Radiotherapy is a good option for patients with positive postoperative margins or postoperative recurrence that precludes a second surgery [9]. A study by Zhao et al. found that the survival of R1 patients who received postoperative adjuvant radiotherapy was comparable to that of R0 resected patients and significantly better than that of the R1 resected patients without radiotherapy [14]. Therefore, R0 resection may not be necessary in patients with a large tumor or a tumor that invades the main bronchus. Postoperative adjuvant radiotherapy can help improve the prognosis of such patients.

After comparing the expression differences between PACC and LUAD in the genome, we obtained a total of 463 upregulated genes and 41 downregulated genes in PACC. The most significantly upregulated genes belong to the trefoil factor family (TFF). The TFF gene family is expressed in lung epithelial cells and plays a crucial role in mucosal protection and repair [15]. TFF proteins maintain or restore respiratory homeostasis by regulating mucus secretion and controlling cellular motility, differentiation, and immune function. This is particularly important in response to tissue damage caused by mechanical injury, harmful chemicals, or pathogens [16], which may also be a factor in the better prognosis of PACC.

In terms of gene mutations, we found that there were significant differences between PACC and LUAD. Of the common driver genes for LUAD, only one of the PACC patients was ALK‐positive, and the rest were undetected, whereas mutations in the NOTCH pathway were more common. This suggests that the targeted therapy may not be useful for PACC. In a previous study, HUO et al. used second‐generation sequencing, Sanger sequencing, and quantitative PCR to detect mutations in common lung cancer driver genes such as EGFR and ALK in 24 PACC patients, and the results showed that all patients had no mutations in common driver genes [17]. Li et al. performed deep sequencing on PACC specimens from eight patients and found that PACC lacked NSCLC driver genes, but had frequent mutations in genes involved in chromatin remodeling and the NOTCH signaling pathway [18]. Conventional targeted therapies may therefore be ineffective for PACC. However, apatinib and axitinib, small‐molecule inhibitors targeting VEGFR, are potentially effective for ACC in a series of small‐sample clinical studies, though their efficacy still needs to be confirmed in subsequent trials [19, 20]. Since ACC commonly has mutations in the NOTCH pathway [21], drugs targeting the NOTCH pathway are emerging. AL101 is also a small‐molecule drug and is a γ‐secretase inhibitor that potently inhibits Notch1–4. Results from preclinical studies using ACC patient‐derived xenografts have shown strong antitumor activity against tumors with Notch‐activating mutations [22]. However, its clinical effectiveness still needs to be verified by further clinical trials. In addition, researchers have found that most ACC cases carry a recurrent chromosomal translocation—t(6;9) (q22‐23;p23‐24)—forming a MYB‐NFIB fusion gene that leads to MYB overexpression [23, 24]. Due to its nature as a transcription factor, MYB was previously considered to be a difficult target. However, with the development of MYB‐targeted inhibitors and the use of MYB‐targeted cancer vaccine therapy, MYB is becoming an increasingly attractive therapeutic target [25].

There are also some limitations to our study. First, the SEER database did not provide detailed information, such as smoking history, R0/R1 resection data, and whether radiotherapy and surgery were palliative or radical treatments; therefore, we could not analyze these variables in our study. Second, the patients were mostly White, which may differ from Asian or Black. In addition, due to the lack of genomic information in the SEER database and the small number of PACC patients in the TCGA database, the clinical information could not be well matched with the genomic data, resulting in bias in the results. Finally, due to the very low prevalence of PACC, our study was retrospective, and it would be difficult to design a more credible cohort study. Research on PACC has been limited to case reports or small case series; thus, our study lacks external validation. It can only be cross‐validated with data from other published articles. We hope that, in the future, large‐scale prospective clinical studies will validate our findings.

## 5 Conclusion

The 3‐, 5‐, and 10‐year OS of the PACC patients was 91.7%, 88.6%, and 85.0%, respectively, compared to 95.8%, 93.9%, and 93.3% for patients undergoing surgery. Race, pathological grade, M stage, regional node exam, and regional node positive were independent prognostic factors for the OS of patients who underwent surgery. Furthermore, our research also assessed the differences in DEGs and mutations between PACC and LUAD. There are significant differences between the genetic map of PACC and LUAD. Additionally, GO and KEGG analyses were used to explore the associated biological characteristics.

## Author Contributions

Z.H., X.J., J.W., Z.C., Q.W., J.Y., L.W., and Z.L. made substantial contributions to the concept and design of the work. Z.H., X.J., Q.S., Y.Y., D.Z., and Z.C. were involved in the acquisition, analysis, and interpretation of clinical data. Z.H., X.J., J.W., Q.S., Z.C., J.Y., L.W., and Z.L. drafted the article and revised it critically for important intellectual content. All authors read and approved the final manuscript.

## Ethics Statement

This study was approved by the Ethics Committee of Zhongshan Hospital, Fudan University, China (B2018‐137R). Informed consent was obtained at the time of hospitalization.

## Consent

We obtained written informed consent from the patient, and the consent is documented in the patient's hospital record.

## Conflicts of Interest

The authors declare no conflicts of interest.

## Supporting information


**Supplementary Figure S1.** Comparison of overall survival between different groups in 167 primary pulmonary and bronchial adenoid cystic carcinoma patients who underwent surgery from the SEER database: T stage, N stage, race, and a nomogram to predict 3‐, 5‐, and 10‐year overall survival of PACC patients who underwent surgery (from top left to bottom right).

**Data S1** Supporting Information.

**Data S2** Supporting Information.

## Data Availability

Detailed information about the 12 patients who underwent surgery at Zhongshan Hospital, Fudan University, is provided in the Supplementary file.
