# Supplementary figures and images for: Analysis of the Clinicopathological Characteristics, Genetic Phenotypes, and Prognostics of Primary Pulmonary and Bronchial Adenoid Cystic Carcinoma
Source: Thorac Cancer. 2025 Jan 19;16(2):e15526. doi: 10.1111/1759-7714.15526 (PMC11742639; doi:10.1111/1759-7714.15526)

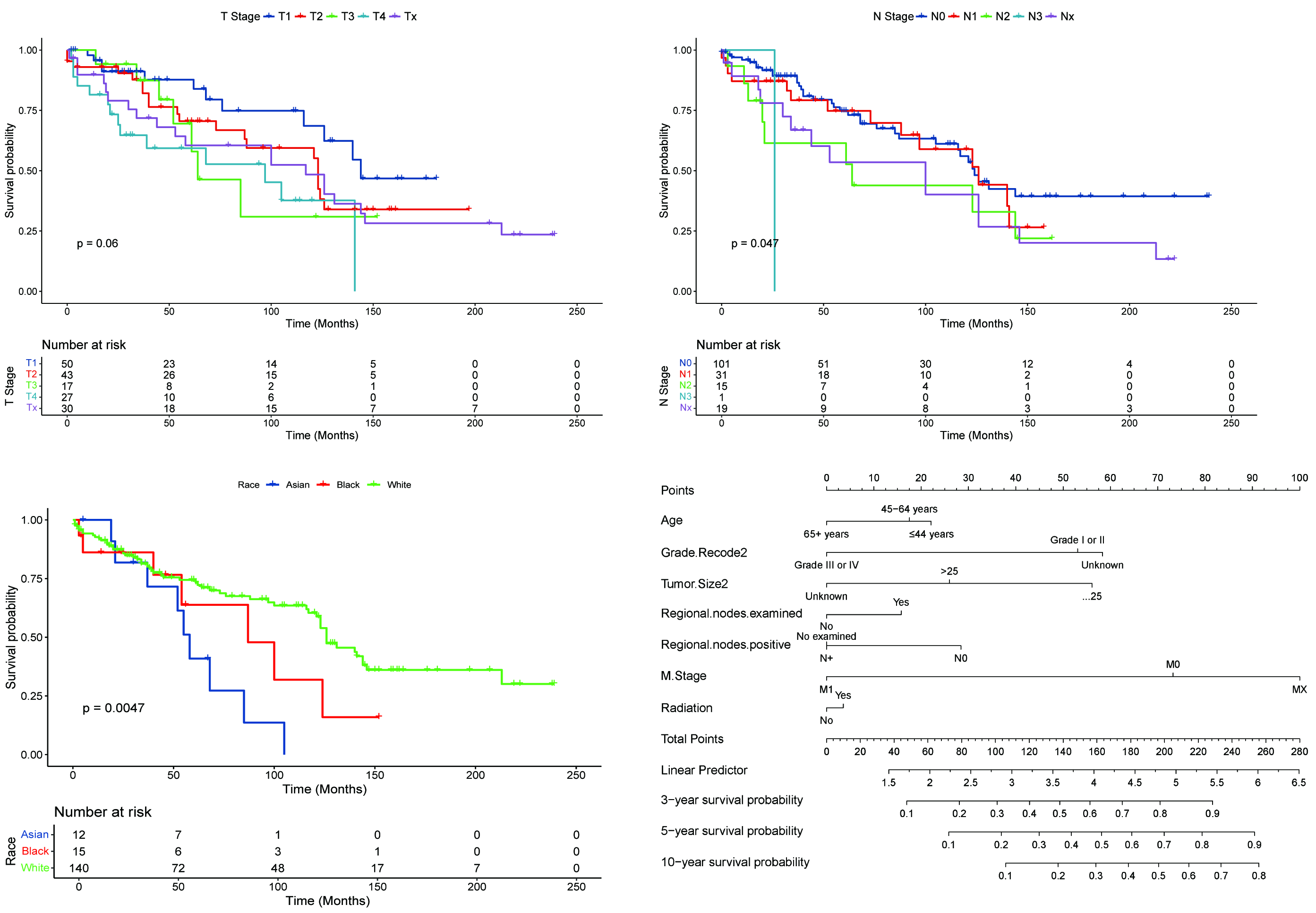

Supplement: Supplementary file 1 — Supplementary Figure S1. Comparison of overall survival between different groups in 167 primary pulmonary and bronchial adenoid cystic carcinoma patients who underwent surgery from the SEER database: T stage, N stage, race, and a nomogram to predict 3‐, 5‐, and 10‐year overall survival of PACC patients who underwent surgery (from top left to bottom right). [file TCA-16-e15526-s003.tif]
